# Health of the South

**Published:** 1843-10

**Authors:** 


### THE WESTERN JOURNAL.

Vol. VIII.—No. IV.

LOUISVILLE, OCTOBER 1, 1843.

### HEALTH OF THE SOUTH.

The public prints inform us that there is a good deal of sickness in some of the Southern States. The yellow-fever prevails in New Orleans, though not in so severe a form, nor so generally as it has in preceding years. At the last dates, however, it was on the increase, and the papers warn the unacclimated to keep away. The country parishes, meantime, are suffering very considerably with bilious fever of a very severe and fatal character. On some of the plantations the distress is said to be extreme.

In Mobile, also, the yellow fever is present, and is of a malignant character.

Vicksburg is represented to be more than usually healthy.

Rodney is suffering greatly. The disease is regarded as a malignant form of bilious fever. Death ensues about the fifth day. C.

